# Microbial biogeography of pit mud from an artificial brewing ecosystem on a large time scale: all roads lead to Rome

**DOI:** 10.1128/msystems.00564-23

**Published:** 2023-09-28

**Authors:** Jun-Lan Mei, Li-Juan Chai, Xiao-Zhong Zhong, Zhen-Ming Lu, Xiao-Juan Zhang, Song-Tao Wang, Cai-Hong Shen, Jin-Song Shi, Zheng-Hong Xu

**Affiliations:** 1 National Engineering Research Center of Cereal Fermentation and Food Biomanufacturing, Jiangnan University, Wuxi, Jiangsu, China; 2 Key Laboratory of Industrial Biotechnology of Ministry of Education, School of Biotechnology, Jiangnan University, Wuxi, Jiangsu, China; 3 National Engineering Research Center of Solid-State Brewing, Luzhou, China; 4 School of Life Science and Health Engineering, Jiangnan University, Wuxi, Jiangsu, China; University of Massachusetts Amherst, Amherst, Massachusetts, USA

**Keywords:** pit mud, bacterial community, spatiotemporal variation, community assembly, co-occurrence network

## Abstract

The fermentation process of strong-flavor *Baijiu* occurs in an underground cellar surrounded by mud, where grains are decomposed and generate new flavor substances. Pit mud microbiome plays a pivotal role in the formation of *Baijiu* flavor, which is positively correlated with cellar age. Previous research on the pit mud microbiome was often conducted for a specific distillery, making it hard to draw a general conclusion to decipher the pit mud microecosystem in such a unique brewing environment. Hence, in this study, we attempt to unravel community formation mechanisms through research in pit mud microbial biogeography based on 12 independent bacterial studies comprising qualified data sets of 302 samples clearly marked with cellar age from five major producing provinces. We found cellar age explained more variations in the bacterial community than geographical region. The bacterial community displayed evident dominant species alternations on a large time scale. *Lactobacillus* was found to be the dominant species in young pit mud (<10 years), while *Caproiciproducens* dominated in aged pit mud. Null model analysis revealed that as pit mud aged, the influence of stochastic and deterministic processes in the bacterial community shifted, and the relative contribution of homogeneous selection increased. Positive correlations dominated the bacterial co-occurrence networks, and the network complexity decreased and stability increased with cellar age. These findings shed new light on the formation of pit mud microbiomes under the impact of long-term batch-to-batch continuous brewing, providing a basis for the evaluation and production of high-quality pit mud.

**IMPORTANCE:**

*Baijiu* is a typical example of how humans employ microorganisms to convert grains into new flavors. Mud cellars are used as the fermentation vessel for strong-flavor *Baijiu* (SFB) to complete the decomposition process of grains. The typical flavor of SFB is mainly attributed to the metabolites of the pit mud microbiome. China has a large number of SFB-producing regions. Previous research revealed the temporal profiles of the pit mud microbiome in different geographical regions. However, each single independent study rarely yields a thorough understanding of the pit mud ecosystem. Will the pit mud microbial communities in different production regions exhibit similar succession patterns and structures under the impact of the brewing environment? Hence, we conducted research in pit mud microbial biogeography to uncover the impact of specific environment on the microbial community over a long time scale.

## INTRODUCTION

Strong-flavor *Baijiu* (SFB), one of the most popular traditional distilled liquors, accounts for 51% of the market share of Chinese *Baijiu*, whose total production based on 65% alcohol content was 7.156 million kiloliters in 2021, according to National Bureau of Statistics data (http://www.stats.gov.cn/english/). Compared with other types of *Baijiu*, SFB employs a mud pit as a fermentation vessel to carry out anaerobic solid-state fermentation with sorghum as the main raw material, which is its most distinguishing feature (1, 2). Pit mud is “alive,” and there are a variety of microorganisms (mainly bacteria and archaea) inhabiting it (3, 4). Recent research has shown that the formation of the representative flavor compounds (e.g., ethyl caproate and ethyl butyrate) of strong-flavor *Baijiu* is attributed to the metabolic activities of microbial communities in pit mud (1, 5), and clostridial microorganisms are further identified as the main producers of caproic acid and butyric acid (6, 7). The structure of the prokaryotic microbial community in pit mud of varying quality differs dramatically, and the relative abundance of *Clostridia* in degraded mud is significantly lower than that in normal and high-quality mud (8), further influencing the accumulation of key flavor compounds such as caproic acid. Enrichment of beneficial functional microorganisms is an important goal in the production of high-quality pit mud. Therefore, exploring the composition and succession patterns of the pit mud microbiome is key to understanding the brewing microecosystem and has practical consequences for the modulation of pit mud quality.

Strong-flavor *Baijiu*-producing regions are widely distributed in China, mainly including Sichuan Province in the southwest, Jiangsu and Anhui provinces in the east, and Henan Province in the central area (Fig. S1). It could be recognized that the microbial communities of pit mud from different distilleries located in different production areas have similar characteristics. The microorganisms in these studied pit mud samples were mainly scattered in the classes *Clostridia*, *Bacteroidia*, *Methanomicrobia*, and *Methanobacteria*, and at the genus level, *Caproiciproducens*, *Syntrophomonas*, *Sedimentibacter*, *Methanosarcina*, and *Methanoculleus* were the predominant populations (3, 8 – 10). Hundreds of years of production experience make people come to a consensus, namely, “the longer the cellar is used continuously, the more likely it is that high-quality *Baijiu* will be produced.” Accordingly, several studies have been conducted on the temporal variations of the pit mud microbiome (3, 4, 9 – 11). The results showed that there were significant differences in the microbial communities of pit mud at different ages, regardless of the production region. The main characteristics were as follows: the relative abundance of *Lactobacillus* in 1-year pit mud had a significant advantage over the other genus (11); however, with the aging of pit mud, the dominant populations in the microbial community gradually changed to microorganisms associated with the enrichment of typical flavor substances of strong-flavor *Baijiu*, such as *Caproiciproducens* (3, 11). In addition, we found that acetic acid and lactic acid, the main products of microbial metabolism in fermented grains (mainly sorghum), were important driving forces for the evolution of the pit mud microbiome (3). As Baas-Becking put it, “everything is everywhere, but the environment selects” (12). Then, will the pit mud microbial communities in different production regions exhibit similar microbial community composition and evolution patterns under the impact of the brewing environment?

Although the preceding research revealed the temporal profiles of the pit mud microbiome from different geographical regions to a certain extent, each separate study focused on pit mud from two or three different age groups from a *Baijiu* producer, preventing a thorough understanding of the pit mud ecosystem. We consider that it is necessary to look at a broader geographical range to explore whether there are similarities and differences in the community structure and succession of pit mud microbial communities in different regions and cellar ages. Microbial biogeography seeks to uncover the mechanisms of microbial diversity maintenance on a spatiotemporal scale using a large sample size, as well as the mechanisms of microbial community assembly in specific habitats (13 – 15). Microbial biogeography is typically concerned with describing the ecological drivers that influence microbial community diversity, such as environmental selection, stochastic drift, diversion, and dispersal limitation (15, 16). Thus, we can better understand the relationship between the environment and the phylogenetic information of microbial communities by elaborating on the contribution of stochastic and deterministic processes to community assembly (17, 18). According to the existing research on the application of microbial biogeography in a wide range of complex habitats (14, 19, 20), in this study, we aimed to reveal the assembly mechanisms and succession patterns of bacterial communities inhabiting a unique brewing ecosystem by studying the geographical characteristics of pit mud bacteria (the most abundant microorganisms) at a large time scale from 1 to 400 years. Moreover, we investigated the correlations and community stability of bacterial communities through co-occurrence network analysis in order to have a deeper understanding of the pit mud microbiota composition and evolution on a large time scale.

## RESULTS

Qualified amplicon data sets of 302 samples clearly marked with cellar age were selected from 12 individual pit mud bacterial studies to explore the influence of geography and age on community diversity and composition (Table S1). Due to the different sequencing regions of the 16S rRNA gene used in these studies, a closed-reference workflow established by Yuan et al. was applied for the identification of operational taxonomic units (OTUs) and taxonomic annotation analysis (21). The core principle of this workflow is to cluster amplicon sequencing fragments into OTUs using complete 16S rRNA sequences in the SILVA database with known taxonomy, which avoids the influence of different primers. The key difference between this workflow and other methods is that it can realize big data analysis in a certain field and solve the problem of analyzing studies with different sequencing primer pairs. We conducted a comparative analysis of the communities among the samples in two dimensions. The samples were classified into four groups on temporal scale, depending on cellar age, including Aa (<10 years), Ab (10–50 years), Ac (~100 years), and Ad (~300 to 400 years); and according to geographical region, the samples could be divided into La (104–106°E), Lb (111–115°E), Lc (115–116°E), and Ld (118–119°E) on the spatial scale (Fig. S1).

### Cellar age contributes more to variations in bacterial diversity than geographical location

**FIG 1:**
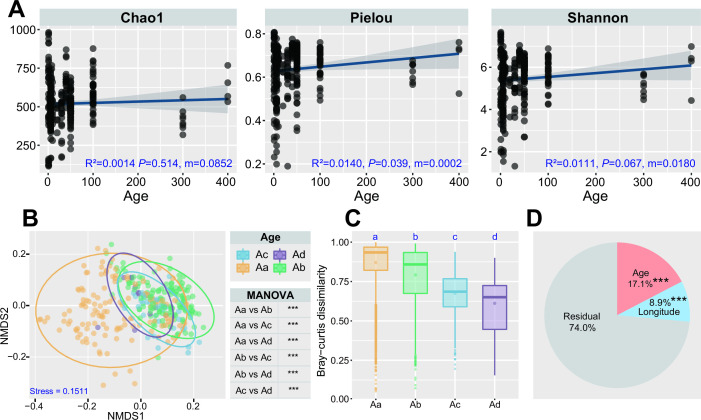
Temporal dynamics of diversity and distribution patterns of pit mud bacteria. (**A**) Statistics and linear regression analysis of α-diversity indices (Chao1, Pielou, and Shannon) of pit mud bacterial communities on a temporal scale. R Square, *P* value, and slope of the fitting lines were respectively marked in each figure. (**B**) Non-metric multidimensional scaling (NMDS) based on weighted UniFrac distance matrices of different age groups. ***P < 0.001. (**C**) Bray-Curtis dissimilarity calculation results of pit mud bacterial community of different age groups. (**D**) The relative contribution of different factors to community dissimilarity calculated via permutational multivariate analysis of variance. Residuals included all factors except Age and Longitude. ***P < 0.001.

A total of 2,511 OTUs was obtained using 97% as a taxonomy identity threshold, and prior to diversity analysis, the sequencing reads of each sample were rarefied to 10,085. Linear regression analysis revealed that the Chao1 richness, Pielou evenness, and Shannon diversity of the pit mud bacterial community increased with cellar age, and in particular, cellar age had a significant (*P* < 0.05) influence on bacterial Pielou evenness (Fig. 1A). We also compared the alpha-diversity indices of the bacterial community in pit mud from different regions at the same cellar age group, and non-significant differences were generally observed, indicating the moderate effect of geographical region on bacterial alpha-diversity (Fig. S2A).

Non-metric multidimensional scaling (NMDS) analysis based on weighted UniFrac distance and multivariate analysis of variance (MANOVA) were performed to evaluate the variations of bacterial communities in pit mud among different cellar ages and geographical regions. NMDS and MANOVA analyses of all 302 samples showed significant differences in bacterial communities in pit mud on a temporal scale (*P* < 0.001) (Fig. 1B). Further investigation revealed that pit mud either with varied cellar ages from the same geographical region or with the same age from different regions also showed significant variances in bacterial communities (Fig. S2B and C). The Bray-Curtis dissimilarity calculation suggested that the difference in bacterial community structure in pit mud significantly decreased as the cellar age increased (Fig. 1C). Furthermore, permutational multivariate analysis of variance (PERMANOVA) analysis proved that cellar age (17.1%) explained more variations in the bacterial community in pit mud than geographical region (8.9%), indicating that cellar age had a greater influence on the formation of the pit mud bacterial community (Fig. 1D).

### The pit mud bacterial community structure dynamically changed during long-term batch-to-batch fermentation

**FIG 2:**
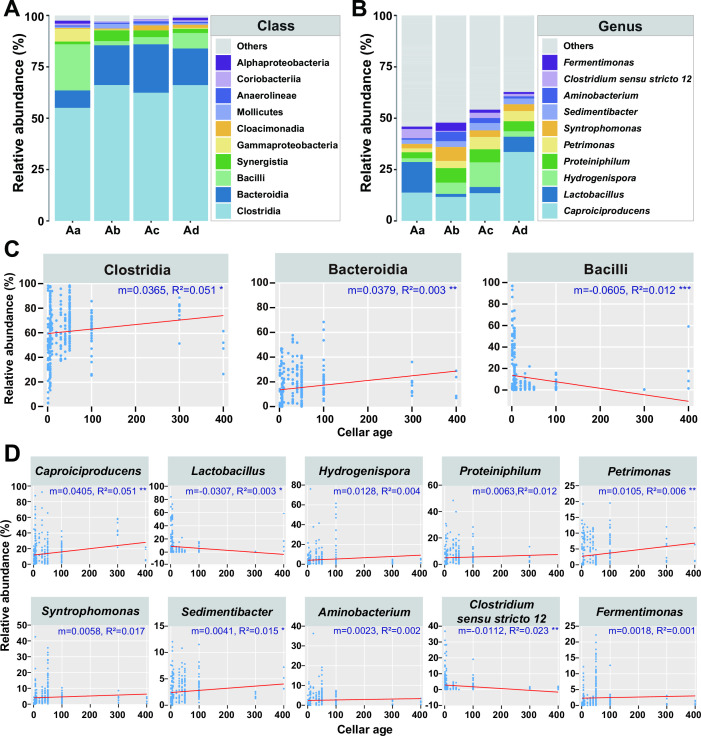
Changes in bacterial community structure in pit mud. (**A**) Relative abundance of the bacterial community in pit mud at the class level on a temporal scale. All other classes were grouped into “others.” (**B**) Relative abundance of the bacterial community in pit mud at the genus level on a temporal scale. All other classes were grouped into others. (**C**) Statistics and linear regression analysis for the relative abundance of the top three classes in all samples on a temporal scale. (**D**) Statistics and linear regression analysis for the relative abundance of the top 10 genera in all samples on a temporal scale. R Square, *P* value, and slope of the fitting lines, respectively, were marked in each figure. *P < 0.05, **P < 0.01, ***P < 0.001.

Following the discovery that cellar age could explain the variations of the bacterial community in pit mud better than geographical regions, greater emphasis was placed on their temporal evolution patterns. At the phylum level, *Firmicutes* had the highest relative abundance across all samples (68.10%–78.08%), showing an absolutely dominant proportion in the bacterial community of pit mud. At the class level, *Clostridia* was the most abundant in all cellar age groups (55.00%–66.13%) (Fig. S3A), and its average relative abundance (ARA) was significantly positively correlated with cellar age (*P* < 0.05), increasing from 55.00% in <10-year samples to 66.07% in ~300- to 400-year samples (Fig. 2A and C). The relative abundance of *Bacteroidia* was significantly increased with cellar age (*P* < 0.05), which was the second predominant class in pit mud samples of greater than 10 years, and its ARA was 19.33%, 20.91%, and 17.83% in the Ab, Ac, and Ad groups, respectively (Fig. 2A). *Bacilli* presented the second highest ARA when cellar age was less than 10 years (Aa, 22.42%), while its relative abundance showed a significantly negative correlation with cellar age (*P* < 0.01), decreasing to 7.52% in ~300- to 400-year samples (Fig. 2A and C). At the genus level, *Lactobacillus* (ARA, 14.88%) was the most abundant in <10-year samples, followed by *Caproiciproducens* (13.75%) (Fig. 2B and S3B). Linear regression analysis demonstrated that the relative abundance of *Lactobacillus* was significantly negatively correlated with cellar age (*P* < 0.05), while *Caproiciproducens* was shown to have a significantly positive correlation with cellar age (*P* < 0.01), making it the genus with the highest ARA in pit mud older than 10 years (Ab, 11.63%; Ac, 16.82%; Ad, 33.53%) (Fig. 2B and D). Furthermore, cellar age was positively correlated with *Hydrogenispora*, *Proteiniphilum*, *Petrimonas* (*P* < 0.01), *Syntrophomonas*, *Sedimentibacter* (*P* < 0.05), *Aminobacterium*, and *Fermentimonas*, and negatively correlated with *Clostridium_sensu_stricto_12* (*P* < 0.01) (Fig. 2D). The similarities and differences of the bacterial communities in different regions were also analyzed. From a holistic perspective, Lc group had the most endemic taxa (22 families), while the Ld group was not identified with endemic taxa at the family level based on the results of Venn diagram (Fig. S4A). Further analysis of different cellar age stages showed similar results that the Lc group held more unique taxa (Fig. S4B and C). From another perspective, the microbial groups shared by samples from different regions included the abundant groups in the pit mud bacterial community, e.g., *Clostridiaceae*, *Rumenococcaceae*, and *Bacillaceae*.

To reveal the possible bacterial biomarkers that correlate with the age of mud cellars, we regressed the relative abundance of the pit mud bacterial community at the OTU level against the age groups of the pit mud using the random forest machine-learning algorithm and adopted a 10-fold cross-validation method with five repeats to evaluate the importance of each OTU (Fig. S5). When the least cross-validation error was found, 65 OTUs were designated as biomarkers in this model (Fig. S5A). These OTUs belonged to 5 phyla, 6 classes, 9 orders, 20 families, and 35 genera. At the genus level, the bacterial community was mainly populated by 10 taxa (average relative abundance >2%). Among these 10 taxa, *Proteiniphilum* was the most abundant genus across all four age groups (13.79%), and its ARA rose drastically from 7.87% to 20.23% with the increase in cellar age (Fig. S5B). The ARA of the OTUs assigned to *Sedimentibacter*, *Caproiciproducens*, and *Syntrophomonas* also showed upward trends with cellar age, increasing from 9.76%, 3.70%, and 1.98% in <10-year samples to 13.96%, 16.88%, and 10.00% in ~300- to 400-year samples, respectively. Conversely, *Clostridium_sensu_stricto_1* and *Clostridium_sensu_stricto_12* had the highest ARA in the <10-year group and decreased sharply with the increase in cellar age, from 31.00% to 5.01% and from 24.62% to 3.93%, respectively.

The linear discriminant analysis (LDA) effect size (LEfSe) method was also used to identify the impacts of cellar age on the pit mud bacterial community, with an LDA score greater than 4 serving as the cutoff (Fig. S6). Seventeen genus-level taxa were discovered as biomarkers for discriminating the pit mud bacterial community between the four age groups. The results revealed that *Lactobacillus*, *Clostridium_sensu_stricto_1*, *Clostridium_sensu_stricto_12*, *Bacillus*, *Garciella*, and *Cupriavidus* were biomarkers of <10-year samples; biomarkers of 10- to 50-year samples included *Syntrophomonas*, *Proteiniphilum*, *Aminobacterium*, *Fermentimonas*, and *Fastidiosipila*; *Caproiciproducens* and *Petrimonas* were the representative genera in ~100-year samples; *Hydrogenispora* and *Ruminofilibacter* were found as biomarkers in ~300- to 400-year samples.

To sum up, considering the results of the above two methods, we found that *Lactobacillus*, *Clostridium_sensu_stricto_1*, and *Clostridium_sensu_stricto_12* presented high relative abundance in pit mud used for less than a decade, suggesting that these might be the biomarker species of this stage. *Syntrophomonas*, *Proteiniphilum*, *Fermentimonas*, *Aminobacterium*, and *Fastidiosipila* were distinguishable marker taxa in aging pit mud (used for several decades). *Caproiciproducens*, *Petrimonas*, and *Hydrogenispora* could be used as marker taxa to distinguish pit mud over 100 years old from those that were not completely aged.

### Bacterial community assembly in pit mud was mainly affected by deterministic process

**Fig 3:**
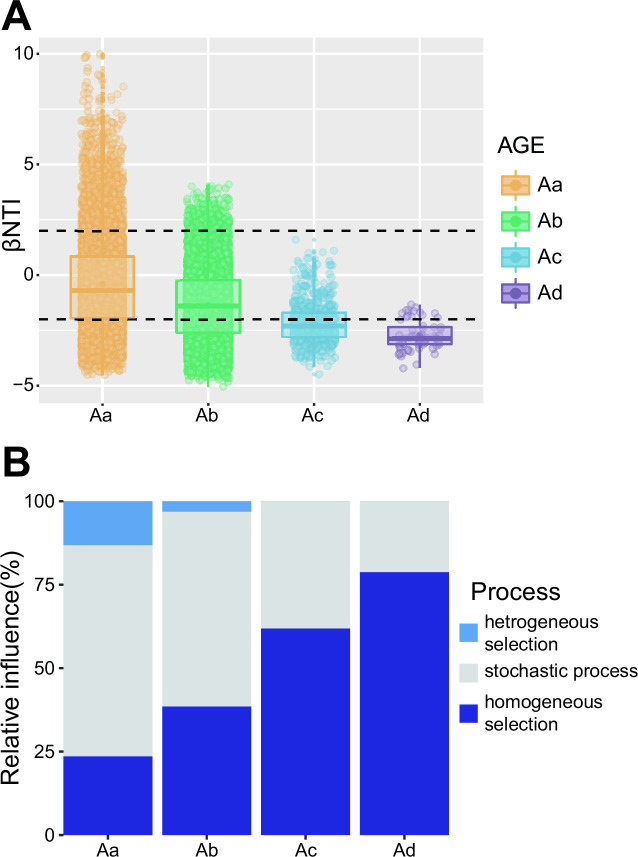
(**A**) Beta-Nearest Taxon Index (βNTI) values of different age groups. (**B**) Relative influence of deterministic and stochastic processes on bacterial community assembly based on the βNTI values on a temporal scale. The βNTI values were calculated via the null model. |βNTI| of ≥2 represents dominant determinism, and |βNTI| of <2 represents dominant stochasticity in driving bacterial community assembly. Moreover, deterministic processes are divided into heterogeneous selection (βNTI >2) and homogeneous selection (βNTI <−2).

According to null model analysis, we assessed the relative influence of stochastic and deterministic processes in pit mud bacterial community assembly by measuring the beta Nearest Taxon Index (βNTI) of each age group (Fig. 3). A null deviation close to zero (|βNTI| < 2) represents that stochastic processes are more important in the assembly process, while large null deviations (|βNTI| > 2) suggest that deterministic processes play a larger role (17). Furthermore, community succession is affected by heterogeneous selection when βNTI is >2 and by homogenous selection when βNTI is <−2. Results showed that the mean βNTI value of the pit mud bacterial community decreased from |βNTI| of <2 to βNTI of <−2 (Fig. 3A), indicating that as pit mud aged, the influence of stochastic and deterministic processes shifted. At the early stage of the aging process of the pit mud (group Aa and group Ab), a high relative influence of stochastic processes was observed in bacterial communities (Aa: 63.26%, Ab: 58.33%), with a low relative influence of deterministic processes (Fig. 3B). As the cellar age increased, the effects of stochastic processes decreased from 63.26% to 21.21%, and the continuously increasing relative contribution of deterministic processes mainly belonged to homogeneous selection (from 23.62% to 78.79%), with the effects of heterogeneous selection decreasing from 13.12% to 0% (Fig. 3B). Collectively, with the aging of pit mud, the bacterial community deviated continuously from the stochastic community model and tended to be consistent with homogeneous selection, which had consistency on a spatial scale.

### The bacterial co-occurrence network complexity reduced and robustness enhanced with cellar age

**Fig 4:**
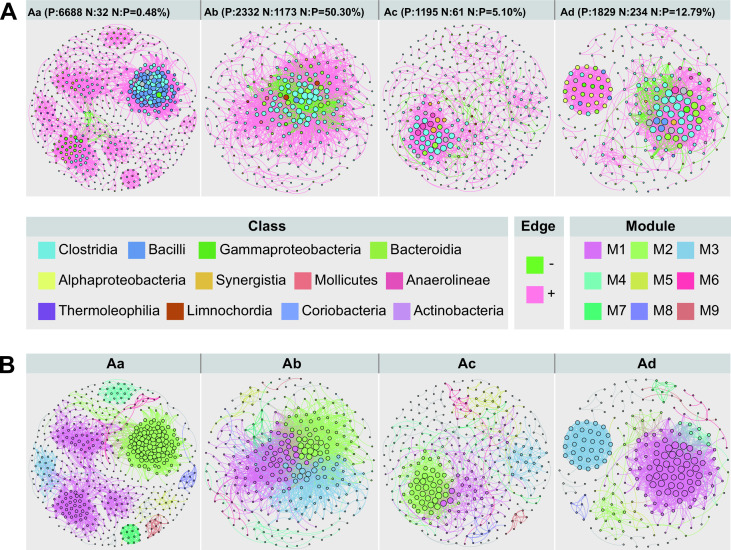
Co-occurrence network analysis of the pit mud bacterial community of the full data set on a temporal scale (Spearman’s *r* >0.7 or *r* <−0.7, *P* < 0.01). The networks were carried out at the class level and presented in the form of microbes (**A**) and modules (**B**) The nodes in panel **A** correspond to those in panel **B.** The size of each node is proportional to the number of connections (degree). (**A**) Each pink edge represents a positive connection; each green edge represents a negative connection.

In order to explore the changes in bacterial interactions in pit mud on a temporal scale, co-occurrence networks were constructed using samples from different cellar age groups based on Spearman correlation coefficients of OTUs (|*r*| > 0.7, *P* < 0.01) (Fig. 4). Our results showed that bacterial network patterns shifted distinctly across four cellar age groups. There were more positive correlations than negative correlations in each of the four co-occurrence networks, and the ratio of negative to positive correlations was 0.48% in Aa, 50.30% in Ab, 5.10% in Ac, and 12.79% in Ad (Fig. 4A). The values of the modularity index of Aa, Ac, and Ad networks were 0.515, 0.525, and 0.472, respectively, suggesting that these networks had a modular structure (>0.4) except group Ab (0.265) (Fig. 4B). The corresponding topological properties of these networks were calculated, respectively (Fig. S7). The number of nodes and edges decreased from 558 (Aa) to 236 (Ad) and from 6720 (Aa) to 2063 (Ad), respectively, as the cellar age increased, indicating that cellar age exhibited a strong impact with a reduction in the total nodes and total edges (Fig. S7A). The network connectivity as measured by the average degree and average weighted degree parameters presented a downward trend from samples less than 100 years to those greater than 100 years (Fig. S7A). Subsequently, we took the La longitude group (Sichuan Province) as an example to examine the temporal variations in the bacterial co-occurrence networks of pit mud samples from the same region of varying ages (Fig. S8). With the increase in cellar age, the number of nodes and edges, the average degree, the average weighted degree, and the density of the network decreased, and the modularity index was higher in networks of pit mud over 100 years (Fig. S7B). In summary, long-term batch-to-batch continuous fermentation reduced the complexity and cohesiveness of pit mud bacterial co-occurrence networks and, at the same time, created a clear division of modules in the network.

**Fig 5:**
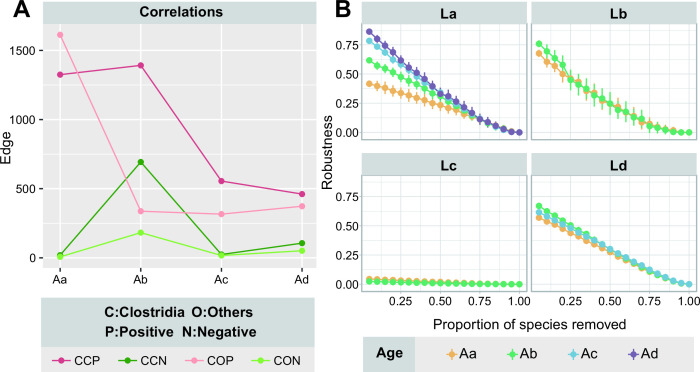
(**A**) Changes in the correlations of *Clostridia* in the co-occurrence network on a temporal scale. The correlations were divided into positive (P) and negative (N) based on the symbiotic relationship between two nodes, and divided into inter-class (CO) and intra-class (CC) based on taxonomy. (**B**) The robustness of co-occurrence networks of different cellar age groups (under the classification of geographical region). Species were removed randomly in a specific proportion (5%).

Since *Clostridia* dominated the pit mud bacterial community and was capable of producing a wide range of key flavor compounds or precursors of Chinese *Baijiu* (e.g., fatty acids), we investigated the correlations between *Clostridia* and *Clostridia* (intra-class) and between *Clostridia* and other classes (inter-class) in the bacterial co-occurrence network of pit mud of different cellar ages (Fig. 5A). The number of positive correlations was obviously higher than the number of negative correlations, whether intra-class or inter-class. As for the internal correlations of *Clostridia*, the positive correlations tended to decrease with the increase in cellar age. The number of negative correlations first increased, then decreased, and finally remained at a low level in the samples >100 years. For the correlations between *Clostridia* and other classes, the number of positive correlations decreased sharply at the early stage of pit mud aging and then remained stable. The number of negative correlations fluctuated and remained at a low level. Comparing intra-class and inter-class correlations, we found that the correlations within *Clostridia* were generally stronger than those between *Clostridia* and other classes. To sum up, *Clostridia* held mainly positive correlations with other bacteria.

The robustness of each network, which was defined as the network’s resistance to node loss after random node removal (22), was calculated to assess the impacts of cellar age on the stability of bacterial co-occurrence networks (Fig. 5B). The robustness of the bacterial co-occurrence network was higher in pit mud samples older than 100 (groups Ac and Ad) than those younger than 50 (groups Aa and Ab). In general, there was no discernible difference in network robustness between samples from groups Aa (<10 years) and Ab (~10 to 50 years). These findings indicated that the stability of the bacterial co-occurrence network in pit mud increased with cellar age.

## DISCUSSION

*Baijiu* is a typical example of how humans employ microorganisms to decompose grains such as sorghum and wheat to create new flavors. As the Chinese proverb says, it is a process of transforming decay into goodness. In addition to water and ethanol, the most abundant flavor substance in strong-flavor *Baijiu* is ethyl caproate. Its precursor (caproic acid) and the precursor of ethyl butyrate (butyric acid) are the main metabolites of pit mud microorganisms (1, 6, 7). A lot of research has shown that under the influence of batch-to-batch brewing production, the microbial community of pit mud was in continuous succession (23, 24). Furthermore, we found that acetic acid and lactic acid produced by grain fermentation were important environmental forces driving this succession (3). Like the division of wine-producing regions, China also has a large number of *Baijiu*-producing regions, each of which has its own unique style. Does the pit mud microbial community influence their uniqueness? What are the similarities and differences between pit mud microbiomes in different production regions? Previous research on the pit mud microbiome was usually carried out for a certain distillery, which made it impossible to fully understand the characteristics of the pit mud microecosystem. Hence, in this study, research in pit mud microbial biogeography was carried out to decipher the composition and evolution of its microbial community in such a unique brewing ecosystem. The general rules of the succession patterns and assembly mechanisms of the bacterial community in the pit mud were summarized in this study, providing a theoretical basis for the evaluation of pit mud quality.

### Replacement of dominant species of the bacterial community in pit mud on a temporal scale

The study proved that the bacterial community of pit mud displayed evident dominant species variations on a large time scale. In this study, *Lactobacillus* was identified as the dominant species in young pit mud (<10 years), while *Caproiciproducens* had a significant advantage in aged pit mud, which was consistent with previous research (3, 10, 24). We further noticed that the patterns previously found on a small scale (e.g., the same batch of pit mud samples or the same distillery samples) were universal across a wide range of regions, indicating that the replacement of dominant species in the pit mud bacterial communities in different production areas was similarly affected by effects whether from the environment or the microbial community itself. Although the environmental factors influencing community succession have already been studied (4), the abiotic and biologic mechanisms that drive community succession remain to be further explored. In view of the possible reasons for the replacement of dominant bacteria, we speculated that it was closely related to the accumulation of nutrients in the cellar. Previous studies found that some bacteria of the family *Ruminococcaceae* (e.g., *Ruminococcaceae* bacterium CPB6) in pit mud prefer to use lactic acid as an electron donor when using substrates for the synthesis of caproic acid production. They also have higher production efficiency in the presence of acetic acid or butyric acid (25, 26). In our previous research, we discovered that lactic acid promoted the accumulation of butyric acid and caproic acid and the enrichment of *Caproiciproducens* more than acetic acid in the simulated fermentation experiment (3). Combined with these conclusions, we believe that the ability to utilize the metabolites of other taxa in the pit mud bacterial community is one of the reasons for *Caproiciproducens*’s gradual rise to the dominant taxon in terms of relative abundance. Notably, the relative abundance of *Lactobacillus* in the late stage of the pit mud aging process was significantly different from that at the beginning. We hypothesized that the environment of pit mud and/or the microbial community exert a niche pressure on *Lactobacillus* and other bacteria that decrease in relative abundance during the aging process of pit mud that is greater than the growth impetus offered to them by the available substrates and other beneficial factors.

In addition, based on the analysis of amplicon data from a wide range of mud samples of different ages, we proposed a new insight into the prediction of mud maturity. There has never been a reliable method for determining the pit mud’s maturity in the manufacturing of strong-flavor *Baijiu*. The maturity of the mud is usually inferred from its smell, color, and the experience of the workers. In this study, through the Random Forests machine-learning algorithm and the LEfSe algorithm, the biomarker taxa of pit mud of different ages were explored, which laid the foundation for the identification method of pit mud of different maturity stages based on community structure. We further found that the biomarkers of pit mud samples at different cellar age stages showed distinct functions. In pit mud used for less than a decade, *Lactobacillus*, the most abundant genus, produces lactic acid and ethanol in the carbohydrate metabolism process (27), providing one of the precursors for the synthesis of caproic acid and influencing the pH of the pit mud environment (11). The biomarkers related to the metabolism of butyric acid included *Syntrophomonas* and *Fastidiosipila*, both of which were marker taxa in aging pit mud used for several decades. As a biomarker of aged pit mud (>100 years), *Caproiciproducens* can utilize glucose, galactitol, or lactic acid to produce ethanol and caproic acid, and it can also be co-cultured with methanogens (28). The biomarkers associated with acetic acid production were *Clostridium_sensu_stricto_12*, *Proteiniphilum*, *Fermentimonas*, *Fastidiosipila*, *Petrimonas*, and *Hydrogenispora* (29 – 34), which could be found in all cellar age stages. Our results indicated that the major functions of the representative bacteria in pit mud changed on a temporal scale.

### The assembly mechanisms of bacterial microbial communities in pit mud are affected by pulse disturbance on a temporal scale

In this study, we investigated the relative contributions of determinism and stochasticity to the assembly of bacterial communities in pit mud. The underlying ecological processes in pit mud bacterial community succession can be inferred by studying the phylogenetic turnover of closely related organisms, where distinguishing between deterministic/niche-driven processes and stochastic/neutral processes is a fundamental framework for understanding community assembly (35, 36). The diversity of microbial communities is shaped by a combination of ecological drivers, including environmental selection, stochastic drift, diversification, and dispersal limitation (15). However, similar studies are still lacking in the study of bacterial community assembly mechanisms in pit mud. Through null model analysis, the relative influence of homogenous selection, heterogeneous selection, and stochastic processes on bacterial community assembly in pit mud were statistically analyzed in this study. Based on previous conceptual models in which stochastic/deterministic equilibrium is coupled to ecological succession (37), this study suggested that the assembly of bacterial communities in pit mud was affected by stochastic processes and changed to homogenous selection on a temporal scale as a result of successive batch-to-batch fermentation impacts. The cellars can be regarded as an artificially created anaerobic fermentation reaction vessel. The long-term batch-to-batch fermentation of Chinese strong-flavor *Baijiu* uses sorghum as its main raw material, which enriches the pit mud with plenty of organic acids, ethanol, and starch (1, 38, 39). These environmental characteristics all exert selective pressure on the assembly of the microbial community in pit mud. Because it had not been continuously intervened in for decades or hundreds of years, the bacterial community’s assembly pattern tended to be stochastic in the early stages of aging. With the continuous application of environmental pressure, the homogeneous selection process continued to affect the assembly of the pit mud microbial community, causing it to be affected by homogeneous selection in the succession process instead.

**Fig 6:**
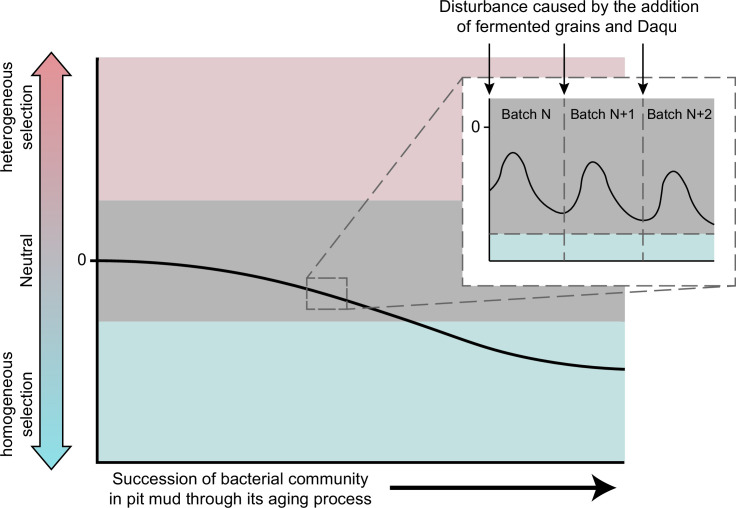
Hypothesis on the succession patterns of bacterial communities in pit mud. The succession of the bacterial community in pit mud was dominated by stochastic processes at the early stage of pit mud aging process and tended to be dominated by deterministic processes belonging to homogeneous selection as pit mud aged. Each addition of new materials caused disruption, but it had no effect on the bacterial community’s overall succession direction.

Based on the characteristics of Chinese strong-flavor *Baijiu* brewing, we further proposed a hypothesis for the change in the assembly pattern of the cellar-mud community (Fig. 6). We hypothesized that the stochastic process triggered by human behavior in continuous batch-to-batch fermentation disrupted the ecological selection pressure given by the pit mud habitat for the bacterial community. Specifically, in between two batches of fermentation, the opening of the cellar, the extraction of the yellow water, and the removal and addition of fermented grains all bring disturbance to the community, causing stochastic processes of birth, death, and extinction, as well as the invasion of new microorganisms into the bacterial community of pit mud, which enhance the influence of stochastic processes on the community assembly patterns (40, 41). Nutrients newly added to the pit can be used as carbon and energy sources by pit mud microorganisms, and the growth of microorganisms utilizing these substrates can enhance random changes in relative population abundance (42). This creates an unstable disturbance to the succession of the mud bacterial community, and we consider it a pulse disturbance rather than a continuous press disturbance (43). Once a new batch of fermentation begins, the environmental impact of the brewing process is restored as metabolites such as alcohol and organic acids accumulate, and the assembly pattern of the community continues to be affected by homogeneous selection after being disturbed by stochastic processes. Such perturbations temporarily changed the effect of ecological processes on the community, affecting its succession rate but not the overall bacterial community’s direction of succession (44). Our inference was consistent with the scenario description of ecological selection in the existing hypothesized conceptual model pointed out in a previous study (37), which proved the rationality of our inference about the succession of the bacterial community in the pit mud. In general, our findings serve as a starting point for further exploration of the succession mechanisms of the bacterial community in pit mud.

### Long-term batch-to-batch fermentation resulted in a simpler and more stable bacterial co-occurrence network in pit mud

The microbial succession process was affected not only by abiotic parameters but also by biotic factors (45). By mapping the bacterial co-occurrence network of samples from different geographical regions and different cellar ages, we investigated the co-occurrence patterns of microorganisms and the stability of the network in order to explore in more details on the succession patterns of pit mud bacterial communities. We found that with the aging of pit mud, bacteria in the pit mud had more simplified correlations on a temporal scale, but the stability of community structure did not decrease with the decrease of connections. There were more correlations between nodes in the bacterial co-occurrence network of young pit mud, and the network was unordered, miscellaneous, irregular, and without clear modules. With the increase of batch-to-batch fermentation cycles, the microorganisms inhabiting pit mud were affected by diverse environmental pressures and complex microbial interactions, and those that were not adapted to the environment would be filtered and die out, ultimately forming a simplified and modular co-occurrence network. This pattern has also been found in metagenomic analysis in our previous study (4). In order to evaluate the stability of the pit mud bacterial community, we examined the robustness of the bacterial co-occurrence network in pit mud of different ages using existing methods (22). In the process of network hubs and nodes decreasing, the stability of the network increased instead of decreasing, which may be due to the decrease of taxa unrelated to key functions and the increase of the relative abundance of key taxa to maintain the stability of the community (46).

We also discovered that after sufficiently long aging of pit mud, taxa dominant in the bacterial community played significant roles in the co-occurrence networks. *Clostridia* was dominant in most of the pit mud samples, and its average relative abundance increased with the aging of pit mud, which was consistent with previous findings (8, 47). Thus, we chose *Clostridia* as the focus group in the network analysis. Previous studies showed that some bacteria (e.g., *Clostridium butyricum*, *Clostridium kluyveri*, and *Ruminococcaceae* bacterium CPB6) belonging to *Clostridia* could produce fatty acids related to the formation of *Baijiu* flavor (26, 48 – 50), and some (e.g., *Clostridium*, *Bacteroides*, and *Ruminococcus*) could also work as hydrolyzers and fermenters to co-metabolize with methanogens and contribute to carbon cycling in anaerobic fermentation systems (8, 49, 51 – 53). Therefore, we focused on the correlations of *Clostridia* in the community in the study of co-occurrence networks. *Clostridia* consistently had more positively correlated connections on the temporal scale, both inter-class and intra-class, which was consistent with the results reported in the previous study, proving that *Clostridia* plays an important role in the succession process of the bacterial community in pit mud (8). Combined with our deduction on stochastic and deterministic processes in the succession of pit mud bacterial communities, we considered that the results of the co-occurrence network support our speculation hypothesis about the succession of pit mud bacterial communities. In the process of continuous batch-to-batch fermentation, some microbial populations with synergistic relationships and possibly shared ecological niches propagate in a stable manner, while populations with excessive negative interaction (competition) gradually lose their ecological niches during community succession. Our results help to reveal the changes in co-occurrence patterns among the bacterial community in pit mud on a temporal scale and provide new insights into the correlation during the succession of pit mud bacterial communities.

### Conclusion

In this study, the composition, succession patterns, assembly mechanisms, and co-occurrence networks of the bacterial community in the pit mud were investigated on both spatial and temporal scales. We found that the influence of temporal factors on the bacterial community in pit mud in different production regions was greater than that of spatial factors, and similar succession patterns were found in the bacterial communities of pit mud in different regions on a temporal scale. Under the influence of continuous batch-to-batch Chinese strong-flavor *Baijiu* brewing activities lasting for several decades or even hundreds of years, the bacteria with the highest relative abundance in the bacterial community changed from *Lactobacillus* in the early aging stage to *Caproiciproducens* in the late aging stage. The bacterial community in pit mud was mainly driven by homogeneous selection and gradually changed from stochastic processes to deterministic processes with the aging of pit mud. The co-occurrence network of the bacterial community in pit mud evolved from complex and multicorrelated in the early stage of pit mud aging to concise and modular in the later stage. These findings promote our understanding of the bacterial community construction and succession patterns in the aging process of pit mud, provide new insights for the future study of artificial pit mud community synthesis, and provide a basis for the evaluation and production of high-quality pit mud. Nevertheless, due to the limitations of data collection, this study did not focus on the influence of environmental factors on the aging process of pit mud. In the future, we will combine the conclusions obtained in this study to further explore the key environmental factors driving the community composition of the bacterial community in pit mud.

## MATERIALS AND METHODS

### Sample collection and description

Forty-eight Chinese strong-flavor *Baijiu* pit mud samples were collected from Suqian (Jiangsu Province) and Luzhou (Sichuan Province), with geographical location and cellar age information recorded. For Suqian samples, we collected eight samples from each cellar that had been continuously used for 10, 30, and 100 years, respectively. For Luzhou samples, we collected six samples from each cellar that had been continuously used for 1, 10, 30, and 100 years, respectively. The samples were frozen, pulverized in liquid nitrogen, and then stored at −80°C for subsequent operations. The DNA extraction, gene amplification, and sequencing methods were the same as those used in previous studies (3). The PowerSoil DNA isolation kit (MOBIO Laboratories, Inc., Carlsbad, CA, USA) was used to extract pit mud DNA, the universal primer pair 338F (5′-ACTCCTACGGGAGGCAGCA-3′)/806R (5′-GGACTACHVGGGTWTCTAAT-3′) was used to amplify the V3-V4 region of the 16S rRNA gene of pit mud bacteria, and the Illumina MiSeq platform was used to carry out paired-end (300 bp × 2) high-throughput sequencing.

The online Chinese strong-flavor *Baijiu* pit mud bacterial 16S rRNA data sets were collected by searching the keywords “strong-flavor *Baijiu*” and “pit mud” in Google Scholar and the National Center for Biotechnology Information (NCBI) SRA database. Twelve studies, comprising 254 bacterial samples with cellar age information, were selected and merged. For all the studies collected online, five studies provided no primer pair information; four studies used V3-V4 or V4 regions to produce amplicons; and two studies used V3–V5 or V4–V5 as amplicon-producing regions. The raw data were obtained from three databases: NCBI, GSA (Genome Sequence Archive), and DDBJ (the DNA Data Bank of Japan). Details of the metadata are provided in Table S1.

After screening, bacterial 16S rRNA metadata of Chinese strong-flavor *Baijiu* pit mud from five different regions was collected from 12 studies for a total of 302 samples. The 302 samples were divided into four groups based on their cellar age (Fig. S1). The pit mud samples aged less than 10 years were classified as group Aa; the pit mud samples aged between 10 and 50 years were classified as group Ab; the pit mud samples aged around 100 years were classified as group Ac; and the pit mud samples aged over 300 years were classified as group Ad. According to geographical variations, the total of 304 pit mud samples can be divided into four groups on a spatial scale (Fig. S1). Samples within the longitude range of 104°E–106°E were classified as group La, while samples within the longitude range of 111°E–115°E were classified as group Lb. The Anhui samples were classified as group Lc (longitude range 115°E–116°E), and samples from Jiangsu were classified as group Ld (longitude range 118°E–119°E).

### Data processing

To deal with sequencing results from different sample sets with different types of primer pairs, we referred to the method of Yuan et al. and adopted the closed-reference workflow approach (21). Paired-end reads were joined by using the fastq_mergepairs command and then trimmed by using the fastq_strip command in VSEARCH (46). After all sequences had been quality-controlled, reads in different 16S rRNA segments were mapped to the full-length 16S rRNA gene sequences using the usearch global algorithm applied in VSEARCH. The database used for taxonomy comparison in this closed-reference workflow (21) was SILVA v.13.2. After the sequences were clustered into OTUs and annotated with taxon information, the results were directly converted to Biological Observation Matrix (BIOM) format using BIOM v.2.1.5 and merged using QIIME (54). The OTU table was filtered following the principle that the minimum count for each sample should be 10,000. The reads of the samples were rarefied to 10,085, and the final number of OTUs was 2,511.

### Statistical analysis

The Chao1, Shannon, and Pielou indices of bacterial communities in pit mud samples were statistically analyzed using the R package “picante” (55), and the honestly significant difference (HSD) test (Tukey’s HSD) (56) was performed using the R package “Multcomp” to verify whether there were significant differences between groups. The β-diversity of the bacterial community was assessed by computing weighted UniFrac distance matrices and visualized by NMDS using the R packages “ape” and “vegan” (57). Significant differences in microbial community structure were performed using PERMANOVA (58) based on the microbial community weighted UniFrac distance metrics and calculated using the R package “picante” and “GUniFrac.” The random forest model (59) and LEfSe analysis (60) were mainly used to identify biomarkers at all classification levels. The random forest algorithm was performed using the R package “randomForest” and LEfSe analysis was carried out using the R packages “coin,” “MASS,” and “rpy2.” To explain the relative influence of stochasticity and determinism on the assembly of bacterial community structure, the βNTI was calculated on both spatial and temporal scale (17). The βMNTD null model was constructed by calculating the mean nearest taxon distance (Ses.MNTD) and the mean nearest taxon distance between communities (βMNTD) by the phylogenetic evolutionary tree and OTU table. The βMNTD was calculated as follows:


$$\beta MNTD=0.5\left[\sum_{i_{k}=1}^{n_{k}}f_{i_{k}}min\left(\Delta i_{k}j_{m}\right)+\sum_{i_{m}=1}^{n_{m}}f_{i_{m}}\min\left(\Delta i_{m}j_{k}\right)\right].$$

The βNTI values were obtained by calculating the difference between each βMNTD sample and the mean value of the βMNTD null model distribution normalized by its standard deviation. The βMNTD was calculated as follows:


$$\beta NTI=\left(\beta MNTD_{obs}\;-\;\bar{\beta MNTD_{null}}\right)/sd\left(\beta MNTD_{null}\right).$$

Then box plots were drawn to determine the driving forces of community phylogeny by the values of βNTI. |βNTI| of >2 was defined as dominant deterministic processes and |βNTI| of <2 as dominant stochastic processes (37). Co-occurrence network analyses were conducted based on Spearman’s correlation scores (Spearman’s *r* > 0.7 or *r* < −0.7; *P* < 0.01). Calculations of network properties were carried out using R (packages “WGCNA” and “igraph”), and network images were generated with topology parameters via Gephi (61) (http://gephi.github.io/), including nodes, edges, average degree, average network distance, average clustering coefficient, modularity index, longest distance (diameter), average weighted degree, density, and connected components. The robustness test and the vulnerability measurement of the network were carried out by R in order to measure network stability (22). The bar charts, pie charts, line charts, box plots, and other plots were visualized by using the package “ggplot2” in R v.4.0.2.

## Data Availability

The raw sequencing data sets have been uploaded to the BIG Sub Genome Sequence Archive (62, 63). The accession number is CRA009373 (BioProject number PRJCA014110).

## SUPPLEMENTAL MATERIAL

Fig. S1 msystems.00564-23-s0001.pdf Main Chinese strong-flavor Baijiu-producing regions in China and the grouping information on a spatial scale.

Fig. S2 msystems.00564-23-s0002.pdf Temporal dynamics of bacterial diversity and distribution patterns in pit mud.

Fig. S3 msystems.00564-23-s0003.pdf Relative abundance of the pit mud bacterial community.

Fig. S4 msystems.00564-23-s0004.pdf Venn diagrams of bacterial communities in pit mud from different regions at the family level.

Fig. S5 msystems.00564-23-s0005.pdf The biomarkers that identified by applying Random Forests machine-learning algorithm.

Fig. S6 msystems.00564-23-s0006.pdf The results of linear discriminant analysis (LDA) effect size (LEfSe) algorithm.

Fig. S7 msystems.00564-23-s0007.pdf The corresponding topological properties of co-occurrence networks of different cellar age groups.

Fig. S8 msystems.00564-23-s0008.pdf Co-occurrence network analysis of bacterial community in group La on temporal scale.

Table S1 msystems.00564-23-s0009.pdf The information of the data sets analyzed in this study.

ASM does not own the copyrights to Supplemental Material that may be linked to, or accessed through, an article. The authors have granted ASM a non-exclusive, world-wide license to publish the Supplemental Material files. Please contact the corresponding author directly for reuse.
