# Supplementary material for: Microbial biogeography of pit mud from an artificial brewing ecosystem on a large time scale: all roads lead to Rome
Source: mSystems. 2023 Sep 28;8(5):e00564-23. doi: 10.1128/msystems.00564-23 (PMC10654081; doi:10.1128/msystems.00564-23)
Supplement: Fig. S1 — Main Chinese strong-flavor Baijiu-producing regions in China and the grouping information on a spatial scale. [file msystems.00564-23-s0001.pdf]

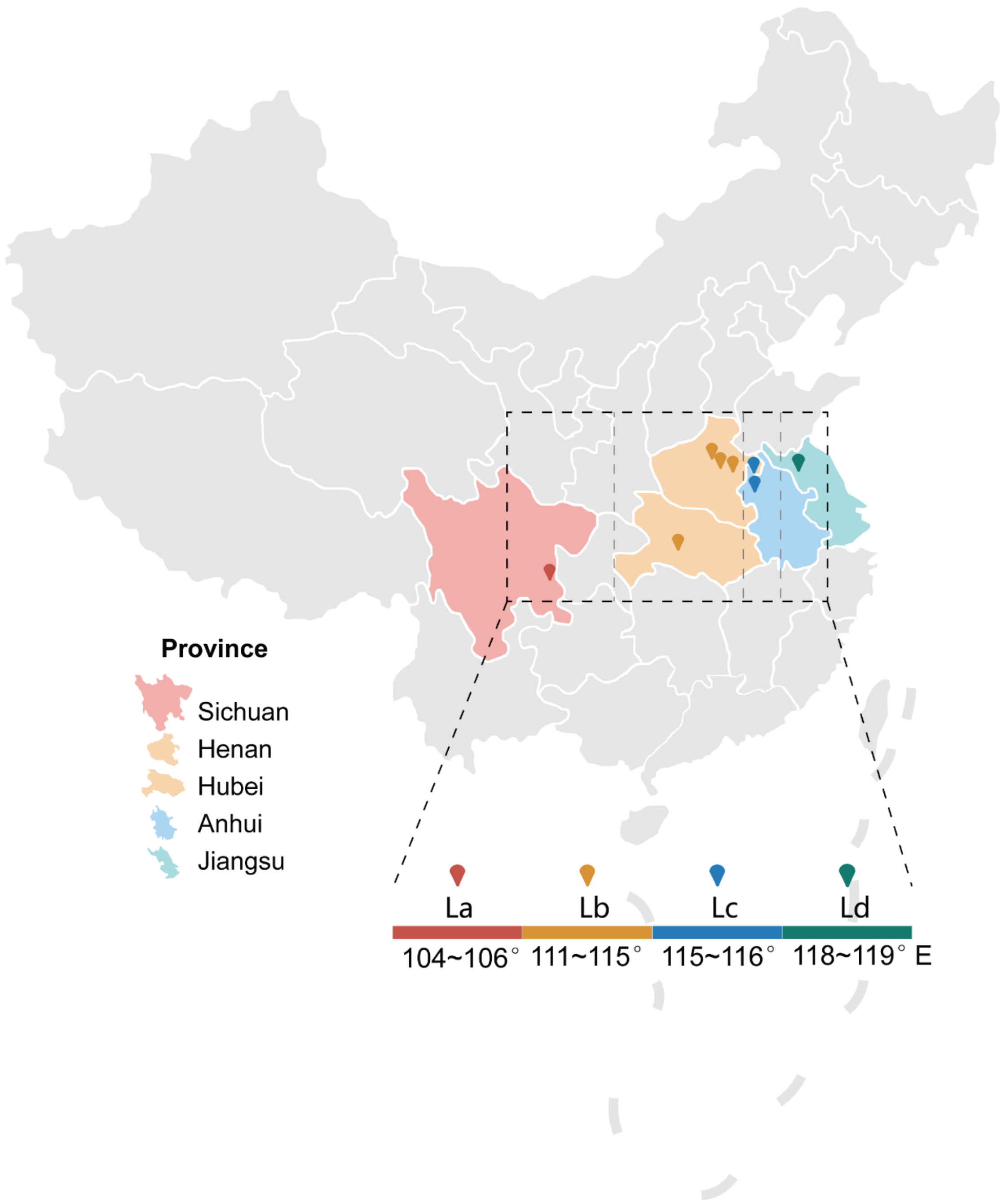

Fig. S1. Main Chinese strong-flavor Baijiu-producing regions in China and the grouping information on a spatial scale.
