# Supplementary material for: Microbial biogeography of pit mud from an artificial brewing ecosystem on a large time scale: all roads lead to Rome
Source: mSystems. 2023 Sep 28;8(5):e00564-23. doi: 10.1128/msystems.00564-23 (PMC10654081; doi:10.1128/msystems.00564-23)
Supplement: Fig. S2 — Temporal dynamics of bacterial diversity and distribution patterns in pit mud. [file msystems.00564-23-s0002.pdf]

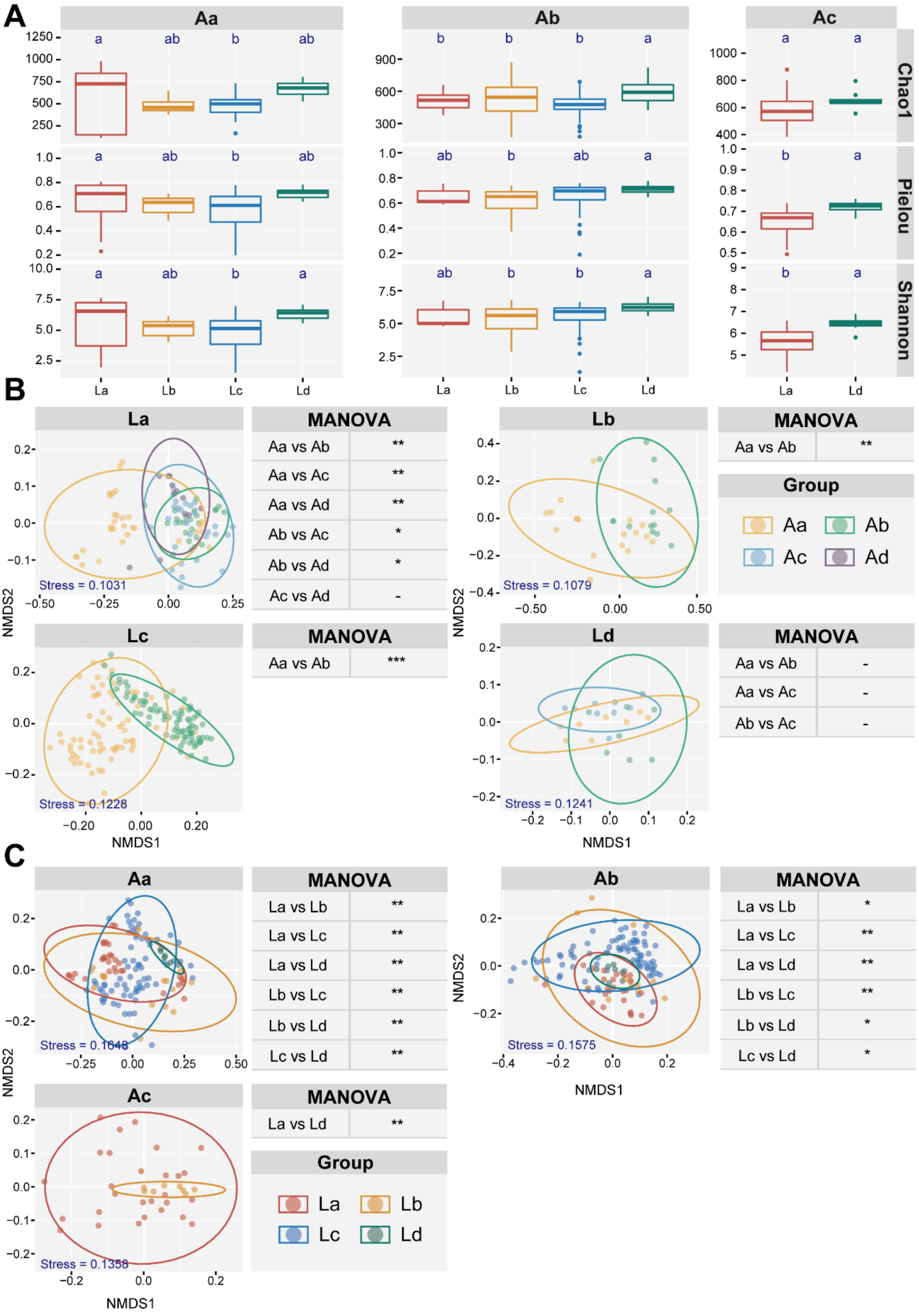

Fig. S2. Supplementary information on the temporal dynamics of bacterial diversity and distribution patterns in pit mud. (A) Chao1, Pielou and Shannon indices for the bacterial community from pit mud samples on a temporal scale. (B) Nonmetric

multidimensional scale analysis (NMDS) based on weighted UniFrac distance matrices of different age groups in different production areas. (C) Nonmetric multidimensional scale analysis (NMDS) based on weighted UniFrac distance matrices of different production areas in different age groups.
