# Supplementary material for: Microbial biogeography of pit mud from an artificial brewing ecosystem on a large time scale: all roads lead to Rome
Source: mSystems. 2023 Sep 28;8(5):e00564-23. doi: 10.1128/msystems.00564-23 (PMC10654081; doi:10.1128/msystems.00564-23)
Supplement: Fig. S3 — Relative abundance of the pit mud bacterial community. [file msystems.00564-23-s0003.pdf]

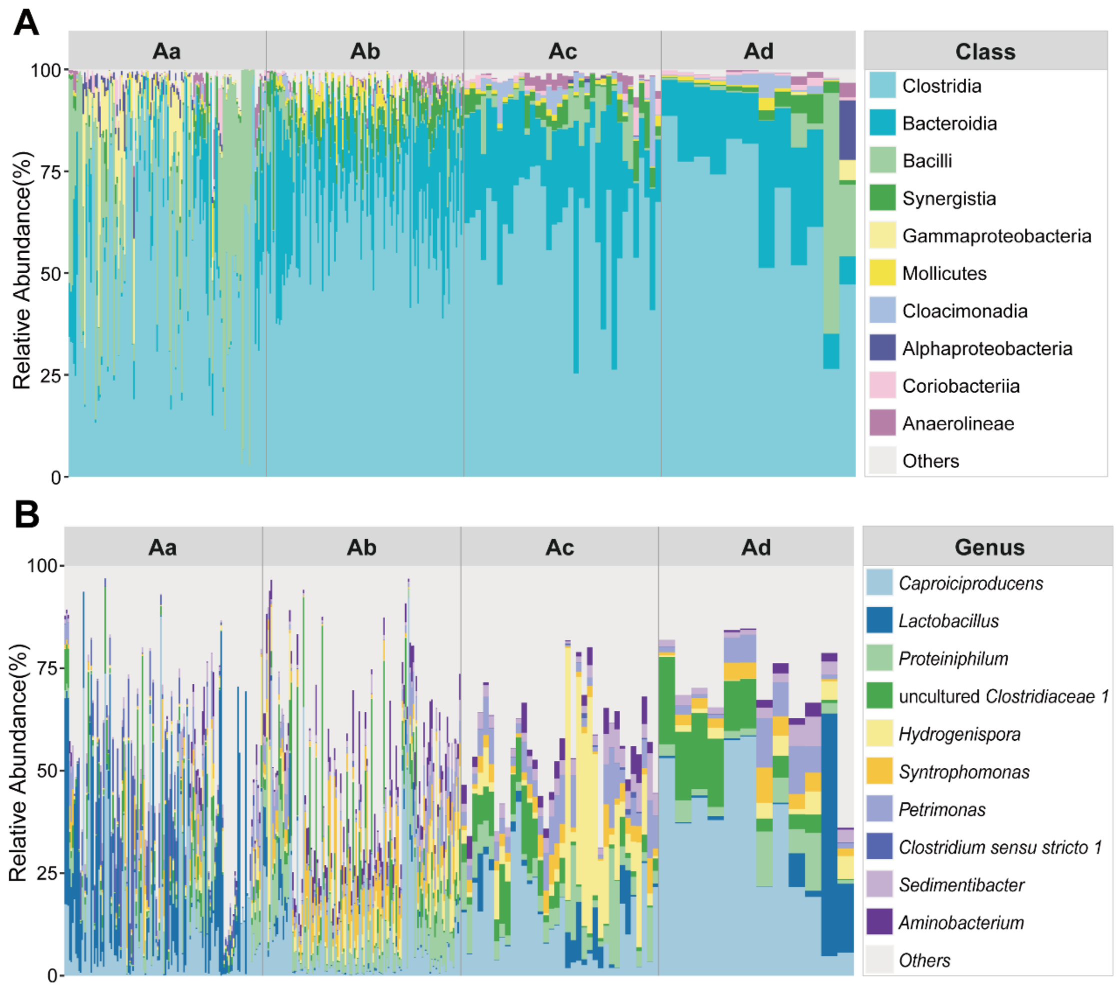

Fig. S3. (A) Relative abundance of the pit mud bacterial community at class level on a temporal scale. (B) Relative abundance of the pit mud bacterial community at genus level on a temporal scale.
