# Supplementary material for: Microbial biogeography of pit mud from an artificial brewing ecosystem on a large time scale: all roads lead to Rome
Source: mSystems. 2023 Sep 28;8(5):e00564-23. doi: 10.1128/msystems.00564-23 (PMC10654081; doi:10.1128/msystems.00564-23)
Supplement: Fig. S4 — Venn diagrams of bacterial communities in pit mud from different regions at the family level. [file msystems.00564-23-s0004.pdf]

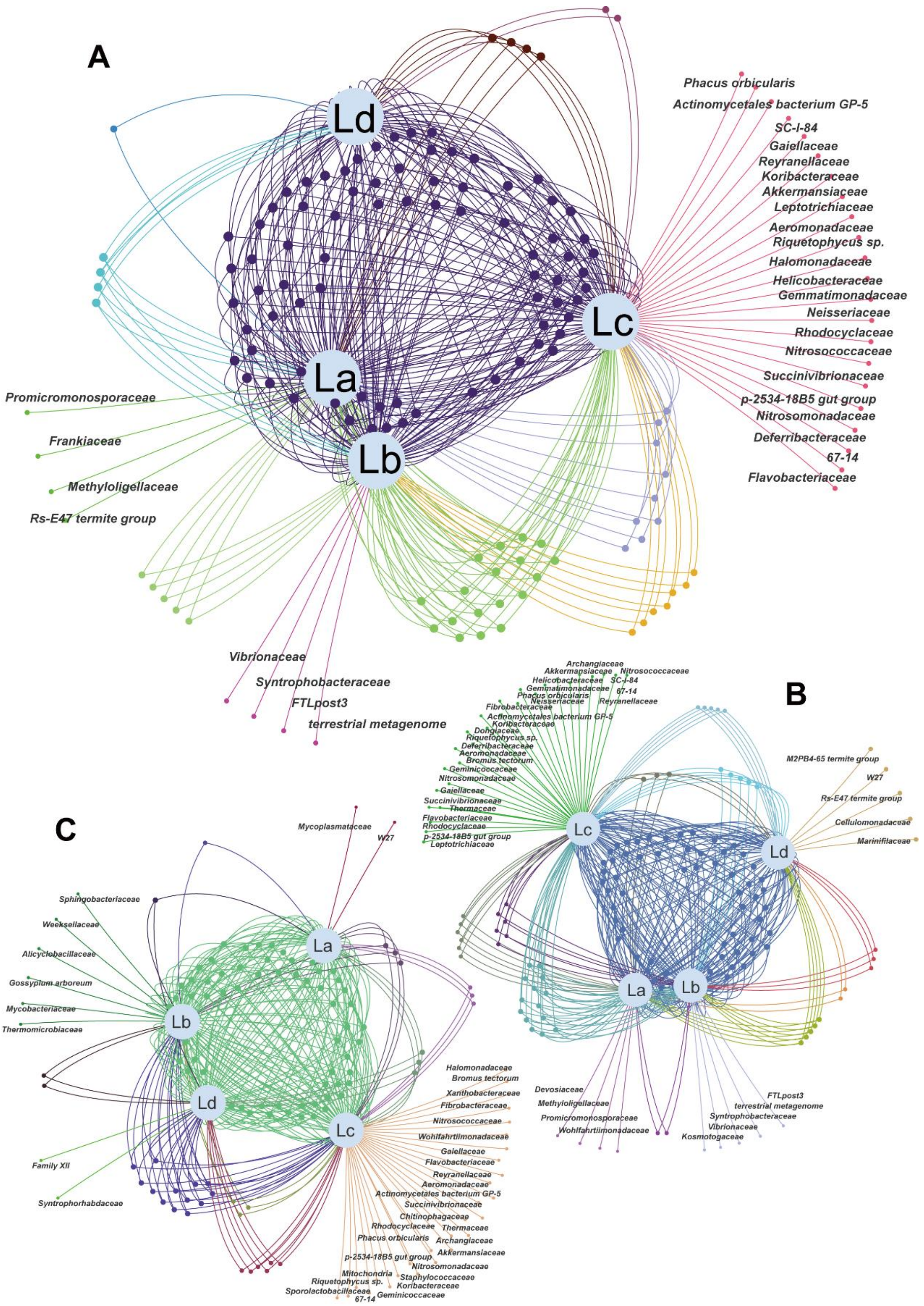


**Fig. S4.** Venn diagrams of bacterial communities in pit mud from different regions at the family level. The diagrams were carried out based on (A) all samples; (B) pit mud used less than a decade (< 10 years); (C) pit mud used for several decades (~ 50 years).
