# Supplementary material for: Microbial biogeography of pit mud from an artificial brewing ecosystem on a large time scale: all roads lead to Rome
Source: mSystems. 2023 Sep 28;8(5):e00564-23. doi: 10.1128/msystems.00564-23 (PMC10654081; doi:10.1128/msystems.00564-23)
Supplement: Fig. S5 — The biomarkers that identified by applying Random Forests machine-learning algorithm. [file msystems.00564-23-s0005.pdf]

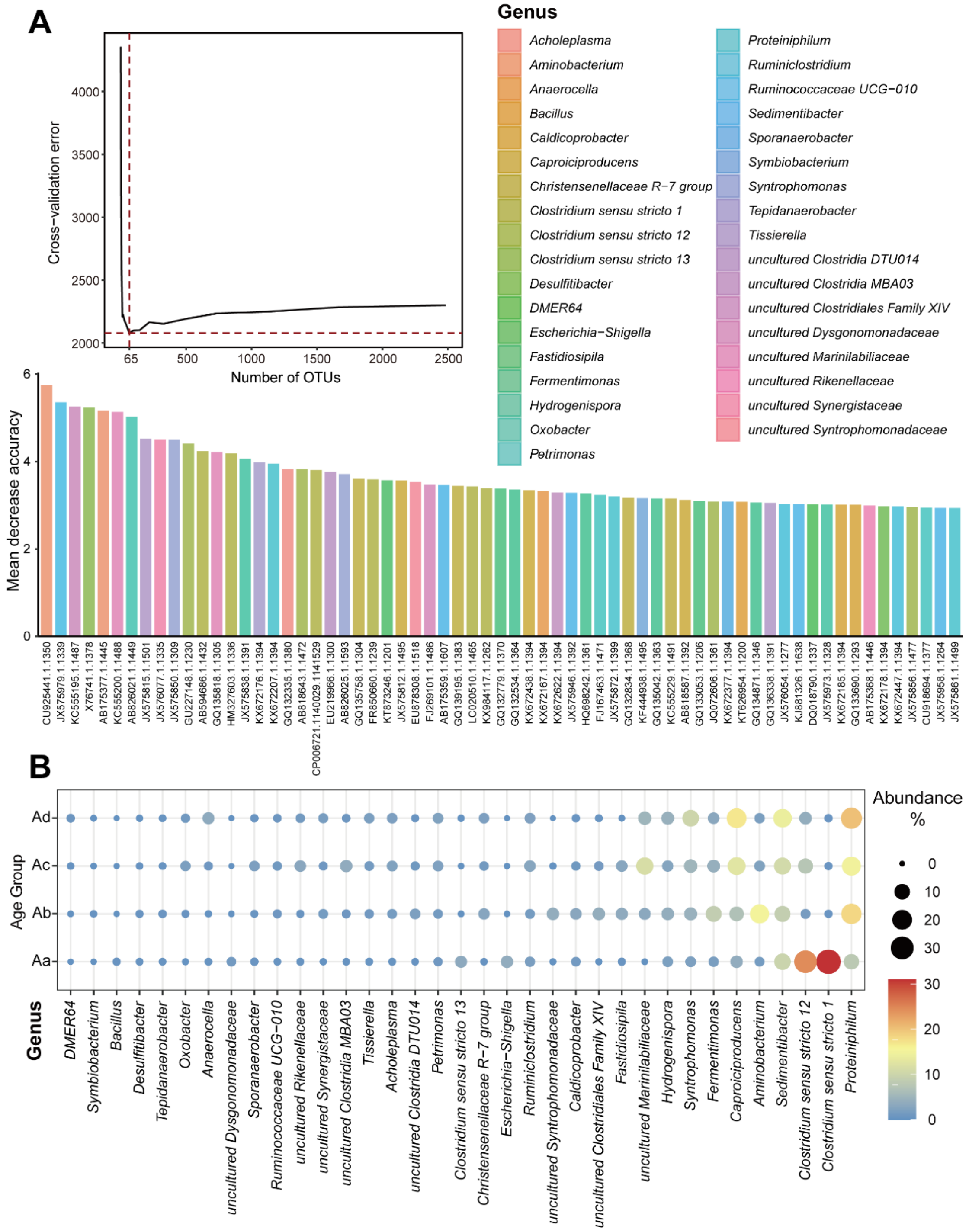

Fig. S5. (A) The biomarkers that identified by applying Random Forests machine-learning algorithm based on their relative abundances. The OTUs were ranked in descending order of importance to the accuracy of the model. The result of the 10-fold cross-validation method was inserted in the figure. (B) Bubble diagram showing the relative abundances of the 35 bacterial genera of different pit mud age groups.
