# Supplementary material for: Microbial biogeography of pit mud from an artificial brewing ecosystem on a large time scale: all roads lead to Rome
Source: mSystems. 2023 Sep 28;8(5):e00564-23. doi: 10.1128/msystems.00564-23 (PMC10654081; doi:10.1128/msystems.00564-23)
Supplement: Fig. S6 — The results of linear discriminant analysis (LDA) effect size (LEfSe) algorithm. [file msystems.00564-23-s0006.pdf]

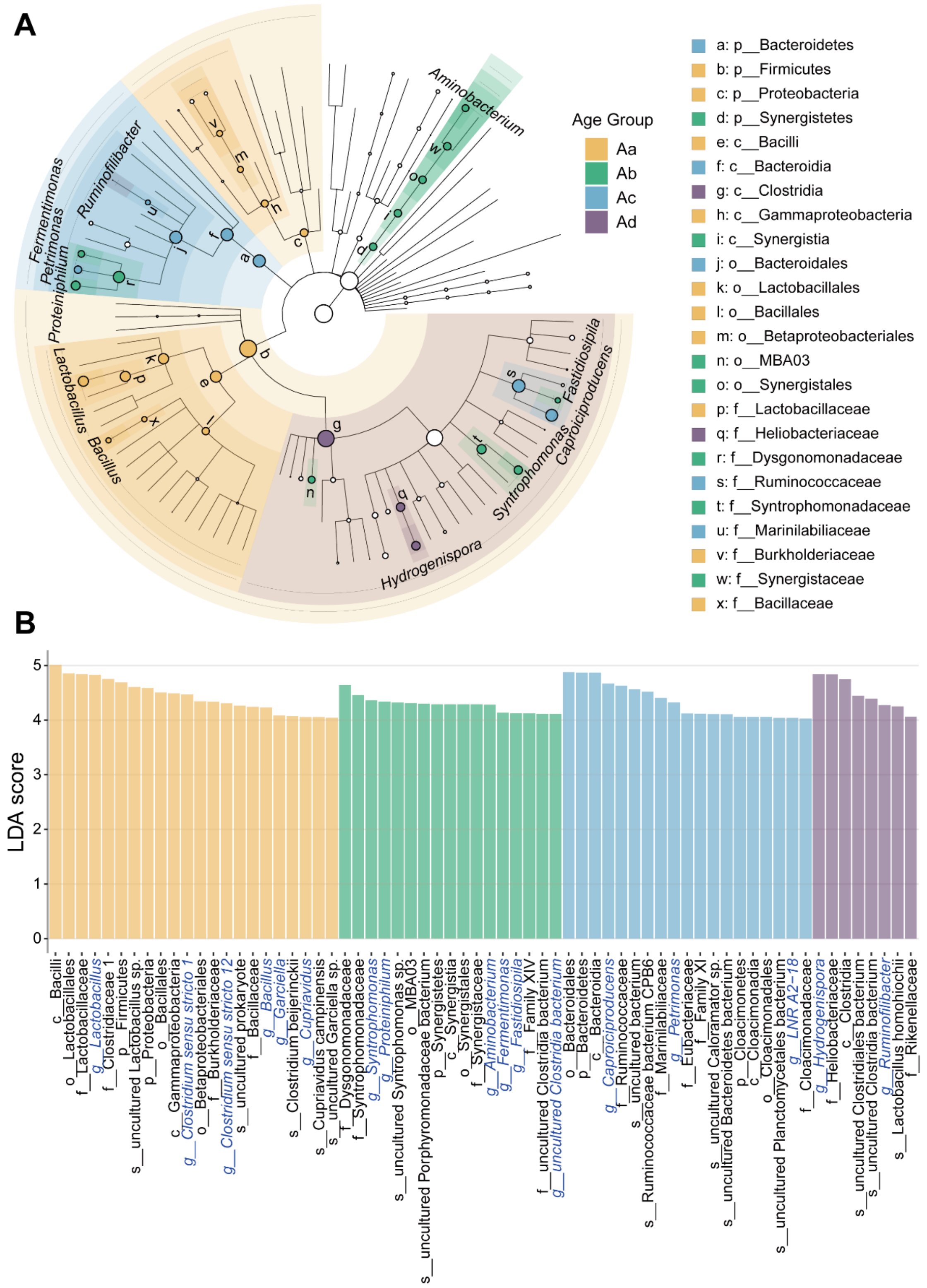

Fig. S6. The results of linear discriminant analysis (LDA) effect size (LEfSe) algorithm. The results of the LEfSe analysis were presented using tree charts (A) and bar charts (B). Genera in the biomarker are labelled blue. The cutoff LDA score was 4.0.
