# Supplementary material for: Microbial biogeography of pit mud from an artificial brewing ecosystem on a large time scale: all roads lead to Rome
Source: mSystems. 2023 Sep 28;8(5):e00564-23. doi: 10.1128/msystems.00564-23 (PMC10654081; doi:10.1128/msystems.00564-23)
Supplement: Fig. S7 — The corresponding topological properties of co-occurrence networks of different cellar age groups. [file msystems.00564-23-s0007.pdf]

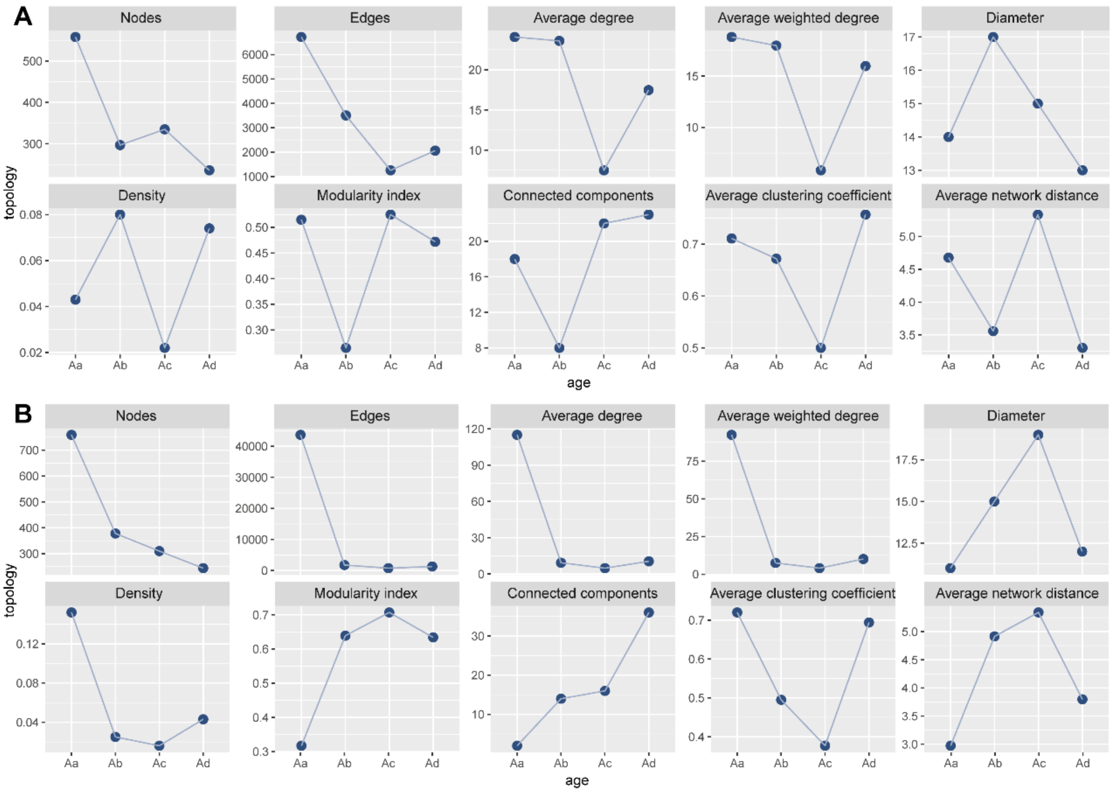

Fig. S7. The corresponding topological properties of co-occurrence networks of different cellar age groups. The topological properties were carried out for all samples (A) and group La (B).
