# Supplementary material for: Microbial biogeography of pit mud from an artificial brewing ecosystem on a large time scale: all roads lead to Rome
Source: mSystems. 2023 Sep 28;8(5):e00564-23. doi: 10.1128/msystems.00564-23 (PMC10654081; doi:10.1128/msystems.00564-23)
Supplement: Fig. S8 — Co-occurrence network analysis of bacterial community in group La on temporal scale. [file msystems.00564-23-s0008.pdf]

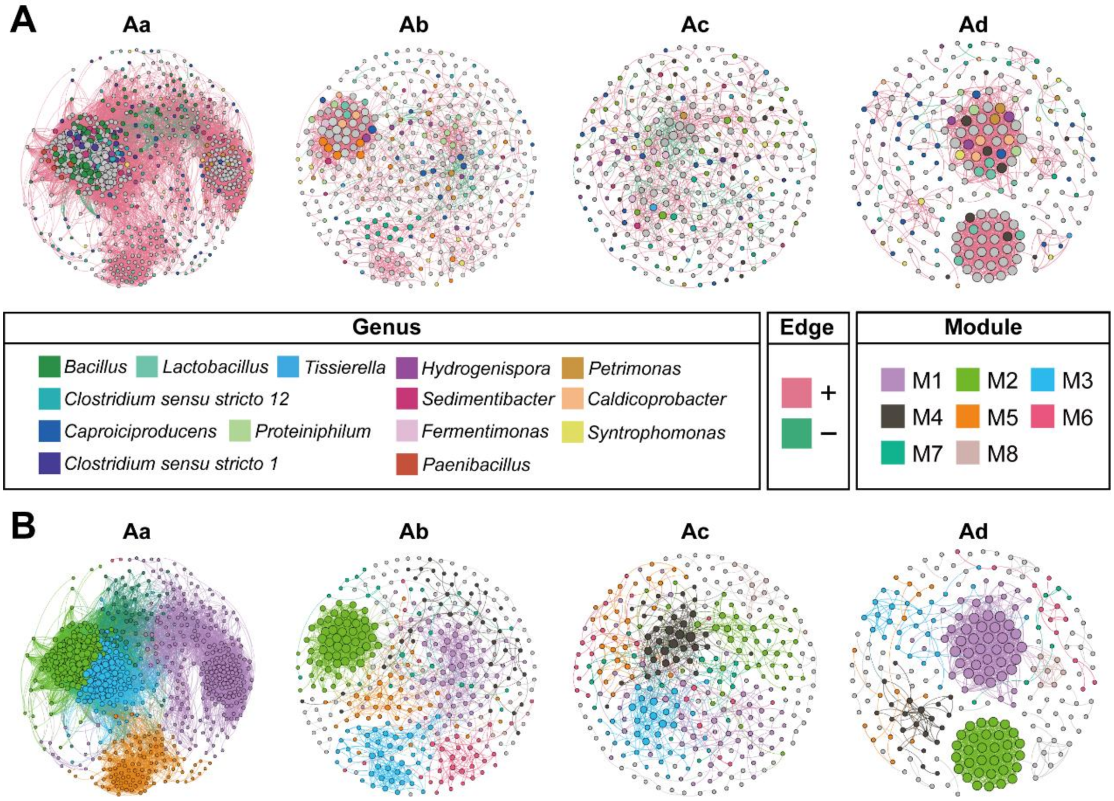

Fig. S8. Co-occurrence network analysis of bacterial community in group La on temporal scale. The networks were carried out on genus level and presented in the form of microbe (A) and module (B).
