# Supplementary material for: Microbial biogeography of pit mud from an artificial brewing ecosystem on a large time scale: all roads lead to Rome
Source: mSystems. 2023 Sep 28;8(5):e00564-23. doi: 10.1128/msystems.00564-23 (PMC10654081; doi:10.1128/msystems.00564-23)
Supplement: Table S1 — The information of the data sets analyzed in this study. [file msystems.00564-23-s0009.pdf]

Supplementary Table 1 The information of the data sets analyzed in this study.

Location	Longitude and Latitude	Sample Size	Sequencing Region	Cellar Age	Data source	Project number	Doi
Anhui-Bozhou	33.50N 116.00E	144	515F-806R	6/50	NCBI	PRJNA540078	https://doi.org/10.1016/j.foodchem.2019.126084
Anhui-Fuyang	32.90N 115.82E	10	missing	20/30	NCBI	PRJNA648265	unknown
Henan-Zhoukou	33.63N 114.63E	9	515F-907R	1/6/10	DDBJ	PRJDB5454	https://doi.org/10.1139/cjm-2020-0012
Henan-Luohe	33.60N 113.70E	16	missing	4/40	NCBI	PRJNA646030	unknown
Henan-Xuchang	34.03N 113.85E	4	missing	1	NCBI	PRJNA669284	unknown
Sichuan-Luzhou	28.53N 105.27E	8	347f-947R	40/400	NCBI	PRJNA389631	https://doi.org/10.1016/j.foodres.2017.09.075
Sichuan-Luzhou	28.87N 105.43E	15	missing	1	NCBI	PRJNA577744	unknown
Sichuan-Luzhou	28.87N 105.43E	9	missing	1	NCBI	PRJNA449898	unknown
Sichuan-Luzhou	28.90N 105.50E	9	338F-806R	100	NCBI	PRJNA558446	https://doi.org/10.1016/j.foodres.2021.110449
Sichuan-Luzhou	28.90N 105.50E	27	338F-806R	30/100/300	GSA	PRJCA003752	https://doi.org/10.1128/AEM.00885-21
Hubei-Zhijiang	31.74N 121.17E	3	341F-806R	10/20/30	NCBI	PRJNA528455	https://doi.org/10.1002/jib.595
Jiangsu-Suqian	33.70N 118.38E	24	338F-806R	10/30/100	This study	PRJCA014110	This study
Sichuan-Luzhou	28.90N 105.50E	24	338F-806R	1/10/30/100	This study	PRJCA014110	This study
